# RalB degradation by dihydroartemisinin induces autophagy and IFI16/caspase-1 inflammasome depression in the human laryngeal squamous cell carcinoma

**DOI:** 10.1186/s13020-020-00340-y

**Published:** 2020-06-19

**Authors:** Xinli Shi, Shenghao Li, Li Wang, Hui Li, Zhen Li, Weiyi Wang, Jing Bai, Yajing Sun, Jianchun Li, Xiaoming Li

**Affiliations:** 1 Department of Otolaryngology Head and Neck Surgery, Bethune International Peace Hospital, Shijiazhuang, 050081 China; 2 Department of Pathobiology and Immunology, Hebei University of Chinese Medicine, Shijiazhuang, 050200 China; 3 Laboratory of Organ Fibrosis Prophylaxis and Treatment by Combine Traditional Chinese and Western Medicine, Research Center of Combine Traditional Chinese and Western Medicine, Affiliated Traditional Medicine Hospital of Southwest Medical University, Luzhou, 646000 China; 4 Department of Neurology, Children’s Hospital of Hebei Province, Shijiazhuang, 050000 China

**Keywords:** Dihydroartemisinin, IFI16 inflammasome, Autophagy, Laryngeal squamous cell carcinoma, RalB

## Abstract

**Background:**

Interferon-inducible 16 (IFI16)/caspase-1 inflammasome activates and secretes IL-1β. However, it is still unclear whether the IFI16 inflammasome is involved in human laryngeal squamous cell carcinoma. Autophagy directly removed inflammasome components and limited early IL-1β production. RalB is required for the crosstalk between inflammasome and autophagy in macrophages. Dihydroartemisinin (DHA), the main derived ingredient of artemisinin, has a variety of biological activities. The mechanism of DHA in regulating the crosstalk between IFI16 inflammasome and autophagy by inhibiting RalB expression was analyzed in order to provide clues for new therapeutic methods in laryngeal cancer.

**Methods:**

The expression of IFI16 was analyzed by Oncomine and GEPIA databases and detected by Western blot and immunohistochemistry. The relationship between IFI16 inflammasome and autophagy was investigated by transmission electron microscopy, immunofluorescence assay, etc. in Hep-2, Cal-27 and HeLa cells treated with DHA. The xenograft tumor of hep-2 cell in nude mice were used to assess the effect of DHA on laryngeal cancer.

**Results:**

It was reported for the first time in this study that IFI16 was overexpressed and positively correlated with caspase-1 in laryngeal carcinoma tissues. DHA significantly inhibited the activation of inflammasome and reduced IL-1β production in the microenvironment of Hep-2 cell xenograft tumor in nude mice. Mechanistically, we found that DHA degraded RalB, inhibited USP33 expression, and triggered autophagy. Meanwhile, enhanced autophagy can reduce the expression of RalB and USP33. Furthermore, DHA promotes autophagy, which suppresses the activation of IFI16/caspase-1 inflammasome and IL-1β production.

**Conclusions:**

Therefore, our findings demonstrate that DHA may act as a RalB inhibitor to regulate the crosstalk between autophagy and IFI16/caspase-1 inflammasome, which inhibits IL-1β production in tumor microenvironment.

## Background

Laryngeal squamous cell carcinoma (LSCC) is a common malignant tumor of head and neck squamous cell carcinoma (HNSCC), accounting for about 5.7 to 7.6% of all HNSCC [1]. The incidence rate has gradually increased in recent years. At present, although surgery remains the main treatment for LSCC, chemoradiation is often needed in advanced and recurrent and/or metastatic cases. However, dose-dependent drug toxicity and tolerance restrict the effect of chemotherapy on laryngeal cancer. Therefore, it is necessary to further explore the molecular mechanism of tumor progression and therapeutic resistance of LSCC, along with the associated new therapeutic strategies.

Long-term inflammatory microenvironment can promote tumor growth. IL-1β is an important molecule involved in the inflammation, carcinogenesis and tumor progression. Some hazardous signals act as the trigger to activate the sensor proteins leading to the formation of inflammasome in the cytoplasm. Inflammasome provides the scaffold to recruit pro-caspase-1, which is cleaved to activate caspase-1, leading to pyroptosis and the production of IL-1β and Il-18 [2]. The pyrin and HIN domain-containing (PYHIN) protein family (for example, absent in melanoma [AIM2], and interferon-inducible 16 [IFI16]) is one of the sensors for inflammasome activation [3]. Recently, it has been found that lower AIM2 expression was negatively correlated with higher p-STAT3 expression in the human hypopharyngeal squamous cell carcinoma (HSCC) tissue samples from 111 patients with HSCC, and combined analysis revealed that the patients with low AIM2 and high p-STAT3 levels had the worst survival rate [4]. IFI16, as a member of PYHIN family, can recognize double strand DNA (dsDNA) from various sources, assemble inflammasome, activate pro-caspase 1, release IL-1β, and induce pyroptosis [5]. However, it is still unclear whether the IFI16 inflammasome is involved in LSCC progression and DHA treatment.

Autophagy directly removed inflammasome agonists and components, limited early IL-1β production, and inhibited the activation of inflammasome [6]. For example, the knockout *of ATG16L1* reduced the secretion of IL-1β in mice [7]. RalB is activated when combined with GTP, thus it can promote the formation of autophagosome [8]. Ras-gene mutation is common in human laryngeal carcinoma. Ras activated three downstream effectors: RAF-MEK-ERK, PI3K-AKT-mTOR and the Ras-like (Ral). Ral is a small GTPase in Ras superfamily. There are two Ral genes of *RALA* and *RALB* in human cells. Elevated expression and activation of Ral was observed in various types of human cancers, regardless of their *RAS* mutation statuses [9]. Targeting of Ras-Ral signaling axis is a potential therapeutic strategy for Ras-driven human cancers. RalB, the key of the Ras-Ral axis, is required for the crosstalk between AIM2 or NLRP3 inflammasomes and autophagy in macrophages [6]. The blockage of RalB would make more important contribution than RAF and PI3K pathways [10]. Therefore, RalB inhibitors represent developing novel agents for cancer therapy [11]. PI3K and RAF inhibitors have already been seen in human cell lines and mouse models [12]. However, the therapies targeting Ras-RalB signaling axis are not available yet.

As an FDA-approved antimalarial drug, dihydroartemisinin (DHA) is the main derived ingredient of artemisinin, which is a natural product from the Chinese herb of *Artemisia annua* L. [13]. DHA is a metabolite produced in the liver from artesunate and artemether, two other artemisinin derivatives [14]. DHA has a variety of biological activities such as anti-inflammation [15], anti-tumor [16] and so on. DHA strongly inhibited virus-induced tumor formation in the oral mucosa of the dogs treated with the canine oral papillomavirus [17]. Our previous studies have confirmed that DHA leads to autophagy and the death of human tongue squamous cell carcinoma (TSCC) cells in vitro and in vivo [18]. Recently, our group showed that DHA induces the activation of AIM2 inflammasome in HepG2215 cells of human hepatocellular carcinoma and autophagy in HeLa cells [19, 20]. DHA has selective toxicity to tumor cells and is likely to become an anticancer drug with low toxicity, high efficiency and low cost [21, 22]. According to the results of the previous work, the mechanism of DHA in regulating the crosstalk between IFI16 inflammasome and autophagy by inhibiting RalB expression was analyzed in order to provide clues for new therapeutic methods in laryngeal cancer.

## Materials and methods

### Cell line and treatment

Human laryngeal carcinoma Hep-2 cells, tongue squamous cell carcinoma Cal-27 cells, cervical cancer HeLa cells were purchased from American Type Culture Collection (Manassas, VA, USA) and cultured in DMEM (Gibco/Thermo Fisher Scientific, Beijing, China) supplemented with 10% fetal bovine serum (Gibco/Thermo Fisher Scientific), 100 U/ml penicillin and 100 μg/ml streptomycin at 37 °C and 5% CO_2_ in an atmosphere of 100% humidity.

DHA (TCI, Japan), etoposide (Sigma-Aldrich, St Louis MO, USA), 3-MA (Sigma-Aldrich) and rapamycin (Sigma-Aldrich) were dissolved in DMSO (Sigma-Aldrich) and stored at -20 °C.

### Bioinformatics prediction

The Oncomine database (http://www.oncomine.com) was used to predict the DNA levels of inflammasome sensors in HNSCC and normal tissues. Then, Gene Expression Profiling Interactive Analysis (GEPIA) (http://gepia.cancer-pku.cn/) was employed to forecast the potential correlation between the expression levels of mRNA in HNSCC.

### Cell viability assay

Hep-2 cells were seeded in 96-well plates (1 × 10^4^ cells/well) and treated with DHA at different concentrations (5, 10, 20 and 40 μM) for 12, 24, 36, and 48 h. Cell viability was determined with Cell Counting Kit-8 (CCK-8, Dojindo Molecular Technology, Japan) according to the manufacturer’s protocol. Finally, optical density (OD) was monitored by a Multiskan Spectrum Microplate Reader (Thermo Fisher Scientific, Inc.) at 450 nm, with 650 nm as the reference wavelength. The cell viability values were calculated as previously described [23]. IC_50_ values were obtained from the cytotoxicity curves using the SOFTmax PRO software.

### Colony formation assay

Hep-2 cells were treated with or without 20.2 μM DHA for 24 h. After treatment, cells were trypsinized and replated into 60 mm dishes at 600 cells per dish. After they were cultured for 14 days, the cell colonies were fixed with chilled methanol, colored by Giemsa staining, and counted under the anatomical microscope. Cloning with a diameter not less than 60 μm is considered a clone.

### Transmission electron microscopy

Hep-2 cells were treated with 20.2 μM DHA for 24 h, then the treated cells were collected, and fixed with 3% glutaraldehyde, postfixed with 1% OsO_4_ (Sangon Biotech), dehydrated in acetone, and embedded in Epon 812 (Nissin EM, Tokyo). Ultrathin sections were stained with 2.0% uranyl acetate/lead citrate, and observed under transmission electron microscope (Hitachi, Ltd., Tokyo).

### Immunofluorescence assay

Hep-2 cells were cultured for 24 h on glass coverslips in 24-well plates (2 × 10^5^ cells/well) with or without treatment with DHA. The samples were fixed, perforated, blocked, and incubated with primary antibody at 37 °C for 1 h and then with the corresponding secondary antibody at 37 °C for 1 h. The primary antibody used in this study included rabbit anti-LC3B antibody (#2775, CST, diluted at 1:400). The used secondary antibodies were Alexa Fluor^®^ 488-conjugated donkey anti-rabbit IgG antibody (Invitrogen Life Technologies, 1:400). Cytoskeleton was stained with phalloidine (Sigma, St Louis, MO, USA) and incubated at 37 °C for 1 h. Cells were counterstained with 4′,6-diamidino-2-phenylindole dihydrochloride (DAPI) (10 μg/ml) (Sigma, USA). Images were captured via a fluorescence microscope (Olympus BX51, Japan), and assessed by confocal microscopy.

### Western blot analysis

These cells were seeded in 6-well plates (3 × 10^5^ cells/well), treated as described above. The whole-cell extracts were directly lysed in SDS sample buffer (50 mM Tris–HCl pH 6.8, 1% SDS, 10% glycerol, 5% β-mercaptoethanol, 0.01% bromophenol blue). The total protein was isolated using RIPA lysis buffer (Solarbio, China) from xenografts in mice, 3 fresh biopsy specimens of laryngeal carcinoma tissues and adjacent normal laryngeal tissues. Protein concentrations were determined by the BCA method. The primary antibodies were mouse anti-IFI16 monoclonal antibody (ab50004, Abcam, diluted at 1:1000), rabbit anti-Caspase-1 antibody (#2225, CST, diluted at 1:500), rabbit anti-IL-1β monoclonal antibody (ab2105, Abcam, diluted at 1:1000), rabbit anti-LC3B antibody (#2775, CST, diluted at 1:400), rabbit anti-Beclin-1 antibody (#3495, CST, diluted at 1:400), rabbit anti-USP33 antibody (ab71716, Abcam, diluted at 1:2000), rabbit anti-RalB antibody (ab129077, Abcam, diluted at 1:1000), rabbit anti-GAPDH polyclonal antibody (#2118, CST, diluted at 1:1000), and rabbit anti-β-actin antibody (BE0021, Bioeasy, diluted at 1:5000). The secondary antibody was goat anti-rabbit IgG-HRP (ZB-2301, ZSGB-BIO, diluted at 1:5000) and goat anti-mouse IgG-HRP (ZB-2305, ZSGB-BIO, diluted at 1:5000). The bands were detected by ECL (enhanced chemiluminescence) detection systems (Vilber, Fusion FX5 Spectra, France). The band intensity was measured by the Image-Pro Plus v6.0 software (Media Cybernetics, USA).

### Patients and tissue specimens

Paraffin-embedded tissue samples from 36 LSCC patients were obtained from the dissected tissues in the archives of the Department of Otolaryngology Head and Neck Surgery, Bethune International Peace Hospital (Shijiazhuang, China) between 2014 and 2015.

The inclusion criteria of the patients were as follows: (i) A definite pathological diagnosis of LSCC; (ii) no anticancer treatment (including chemoradiotherapy or biotreatment) before laryngectomy; (iii) the absence of common diseases such as diabetes, hypertension, coronary heart disease (CHD), and no history of long-term drug use; (iv) the availability of formalin-fixed, paraffin-embedded tissues; and (v) the availability of complete clinicopathological and follow-up data.

The 36 HSCC patients were aged from 31 to 79 years with the mean age of 58 years. The clinicopathological characteristics of the HSCC patients are summarized in Table 2. The clinical stage of tumors was evaluated on the basis of the laryngeal cancer staging system of the American Joint Committee on Cancer (AJCC) in 2017. The surgical procedures for local LSCC involved the resection of tumor with or without the preservation of laryngeal function.

The study was approved by the Medical Ethics Institute of Bethune International Peace Hospital (Permit number: 2017-KY-02). All the samples were anonymous. Moreover, the fresh tissue specimens from 3 LSCC and the corresponding adjacent normal laryngeal tissues were collected for Western blotting at our institute in 2016. The corresponding adjacent normal laryngeal tissue with a 0.5 cm of cancer resection margin was selected during the surgery. Postoperative pathology confirmed that this tissue was noncancerous.

### Establishment of xenograft tumors and treatment of animals

Female BALB/c nude mice (Vital River Laboratory Animal Technology Co. Ltd., Beijing) at the age of 5–6 weeks were used. Each mouse was subcutaneously inoculated with 1 × 10^7^ Hep-2 cells in the left inguinal area to establish the xenograft tumor. When the average tumor size reached 5 mm in diameter, the tumor-bearing mice were randomly distributed into four different groups with six animals in each group. The mice in the DHA group received intraperitoneal injection of DHA in DMSO (25 mg/kg), once daily for five consecutive days per week for 21 d. The mice in the DDP group were intraperitoneally injected with 2 mg/kg Cisplatin (Sigma-Aldrich, 15663-27-1) once every 2 days [24]. The mice in the normal control (NC) group were intraperitoneally injected with 0.1% DMSO in physiological saline. The tumor size and body weight of each animal were measured every 5 days throughout the study. Tumor volume was calculated by the formula: V (mm^3^) = width^2^ (mm^2^) × length (mm) × 0.5. The inhibition rate of tumor growth was calculated by the formula (1-the average tumor weight of the experimental group/the average tumor weight of NC group) × 100%. During the treatment, no mice died from loading tumor. After 21 days of treatment, all the animals were sacrificed by cervical dislocation at the termination of experiments, and the tumors were removed, weighed, fixed in 4% paraformaldehyde, and embedded in paraffin.

All the animals were maintained in SPF facility with the constant temperature of 22–24 °C and a dark–light cycle of 12 h/12 h, and housed in plastic cages. The protocol was approved by the Ethics Committee for Animal Experiment of Bethune International Peace Hospital (Permit number: 2017-KY-18).

### Immunohistochemistry (IHC)

For histological examination, all the paraffin-embedded tissue samples were cut into 4 μm serial sections on glass slides, baked at 70 °C for 15 min, and dehydrated with gradient ethanol. Then, antigen was retrieved by heating to 121 °C for 3 min in 10 mmol/l citrate buffer (pH 6.0) with an autoclave. After the endogenous enzyme was inactivated by hydrogen peroxide (0.3%), the sections were incubated with normal goat serum at room temperature for 15 min, and incubated with mouse anti-IFI16 monoclonal antibody (ab50004, Abcam, diluted at 1:200), rabbit anti-Caspase-1 antibody (#2225, CST, diluted at 1:200), or rabbit anti-IL-1β monoclonal antibody (ab2105, Abcam, diluted at 1:200) overnight at 4 °C. PBS was used as the negative control for the primary antibody. The sections were rinsed with PBS for 3 times, and then incubated with the secondary antibody at 37 °C for 45 min. After they were rinsed with PBS, the sections were developed with 3, 3-diaminobenzidine (DAB) kit (ZLI-9018, ZSGB-BIO, China) for 5–10 min, and washed with tap water. Next, the sections were counterstained with hematoxylin, desalinated by dilute hydrochloric acid, and rinsed for 5 min. Subsequently, the sections were dehydrated, cleared, mounted, and examined with a microscope. The results of immunohistochemistry were examined by 2 senior histopathologists using the double blind method. The cytomembrane/cytoplasm stained with light yellow or tan were regarded as positive cells.

IHC staining was scored according to the following method: according to the staining intensity of immunohistochemistry, negative was scored as 0 point, light yellow as 1 point, moderate yellow as 2 points, and tan as 3 points. The percentage of positive cells in total cells of ≤ 5% was scored as 0, that of 6–25% was scored as 1 point, that of 26–50% was scored as 2 points, and that of > 50% was scored as 3 points. The judgment of protein expression is based on both the staining intensity and positive cell rate, and the product of these two values was calculated. After the multiplication of the two scores, they were divided into two groups: the group with the product of not less than 3 points was defined as the high expression group, and the group with the product of less than 3 points was defined as the low expression group.

### Statistical analysis

All statistical tests were performed by SPSS19.0 statistics software (SPSS, Chicago, IL). All in vitro experiments were repeated for at least three times. The data were presented as mean ± SD. When more than two groups were enrolled, the mean values were compared between each two groups with one-way ANOVA or student’s t test. The IFI16 and caspase-1 expressions in laryngeal carcinoma tissues and that of IL-1β protein in xenograft tumor from mice were analyzed by Pearson’s χ^2^ test. The difference with *P *< 0.05 was considered statistically significant.

## Results

### Gene expression and transcription levels of inflammasome sensors in HNSCC patients

The sensors for inflammasome activation can be classified as members of PYHIN protein family or the NOD-like receptor (NLR) protein family (For example, NLRP1, NLRP3, NLRC4, NLRC5, NLRP6, NLRP12, NOD1 and NOD2). Bioinformatics analysis was conducted to screen the expression difference of these molecules. Oncomine database (http://www.oncomine.org) is the largest oncogene chip database and integrated data mining platform at present. First, the differential gene expressions of 10 sensor genes and 2 key molecules of the pathway in TCGA Head-Neck database were investigated (n = 364).

The expression of two ALRs (*IFI16* and *AIM2*) and three (*NLRP3*, *NLRC4* and *NOD1*) of eight NLR genes was significantly upregulated in 290 HNSCC when compared with that in 74 normal controls (*P *< 0.05, Table 1). Two ALRs (*IFI16 and AIM2*) and three NLR genes (*NLRP3*, *NLRC4* and *NOD1*) were changed by less than twofold in gene expression levels (Table 1). No significant difference in the expression of the NLR genes (*NLRP1*, *NLRP6*, *NLRP12*, *NOD2*) was observed in TCGA Head-Neck database (*P *> 0.05, Table 1).

**Table 1:** Analysis of differences in expression levels of inflammasome related molecules in HNSCC by Oncomine Database

	Head–neck carcinoma vs. normal			Head–neck carcinoma vs. buccal mucosa			Head–neck carcinoma vs. normal		
	TCGA head–neck database (n = 290)			Ginos head–neck database (n = 54)			Cromer head–neck database (n = 38)		
	Fold change	P-value	t-test	Fold change	P-value	t-test	Fold change	P-value	t-test
*IFI16*	1.048	1.71E−11	6.889	3.115	9.22E−15	13.011	1.435	0.17	1.117
*AIM2*	1.048	1.71E−11	6.882	5.674	6.51E−14	11.201	2.028	0.001	3.912
*NLRP1*	− 1.035	1	− 4.674	1.674	0.037	1.878	1.089	0.428	0.192
*NLRP3*	1.053	3.73E−12	7.133	1.4551	0.035	1.879	1.555	0.192	0.976
*NLRC4*	1.033	2.02E−08	5.633						
*NLRC5*	1.001	0.443	0.144						
*NLRP6*	− 1.064	1	− 7.355						
*NLRP12*	− 1.017	0.994	− 2.53						
*NOD1*	1.08	1.64E−19	9.62	2.322	1.12E−04	5.034			
*NOD2*	− 1.005	0.747	− 0.664	− 1.72	1	− 3.866			
*CASP1*	− 1.079	1	− 6.227	1.445	4.26E−04	3.548	1.325	0.215	0.869
*IL1B*	1.028	2.40E−06	4.661	2.801	1.77E−08	6.605	4.908	0.002	5.574

The findings in TCGA Head-Neck database were confirmed in Ginos Head-Neck database (n = 54) with 41 HNSCC and 13 Buccal Mucosas. It was found that the mRNA expression levels of *IFI16*, *AIM2*, *NLRP3*, and *NOD1* were significantly upregulated in Ginos Head-Neck database (n = 54) (*P *< 0.05, Table 1). ALRs (IFI16 and AIM2) were more pronounced (> twofold) than NLR (*NLRP3* and *NOD1*) (Table 1). Moreover, the expression levels of IFI16 (> threefold) and AIM2 (> fivefold) were increased to a much greater extent than those of NLRP3 (1.4-fold) and NOD1 (2.3-fold) in HNSCC versus the control buccal mucosas (Table 1). Similarly, the mRNA expression levels of *CASP1* and *IL1B* were significantly upregulated in Ginos Head-Neck database (n = 54) (*P *< 0.05, Table 1).

**Fig. 1:**
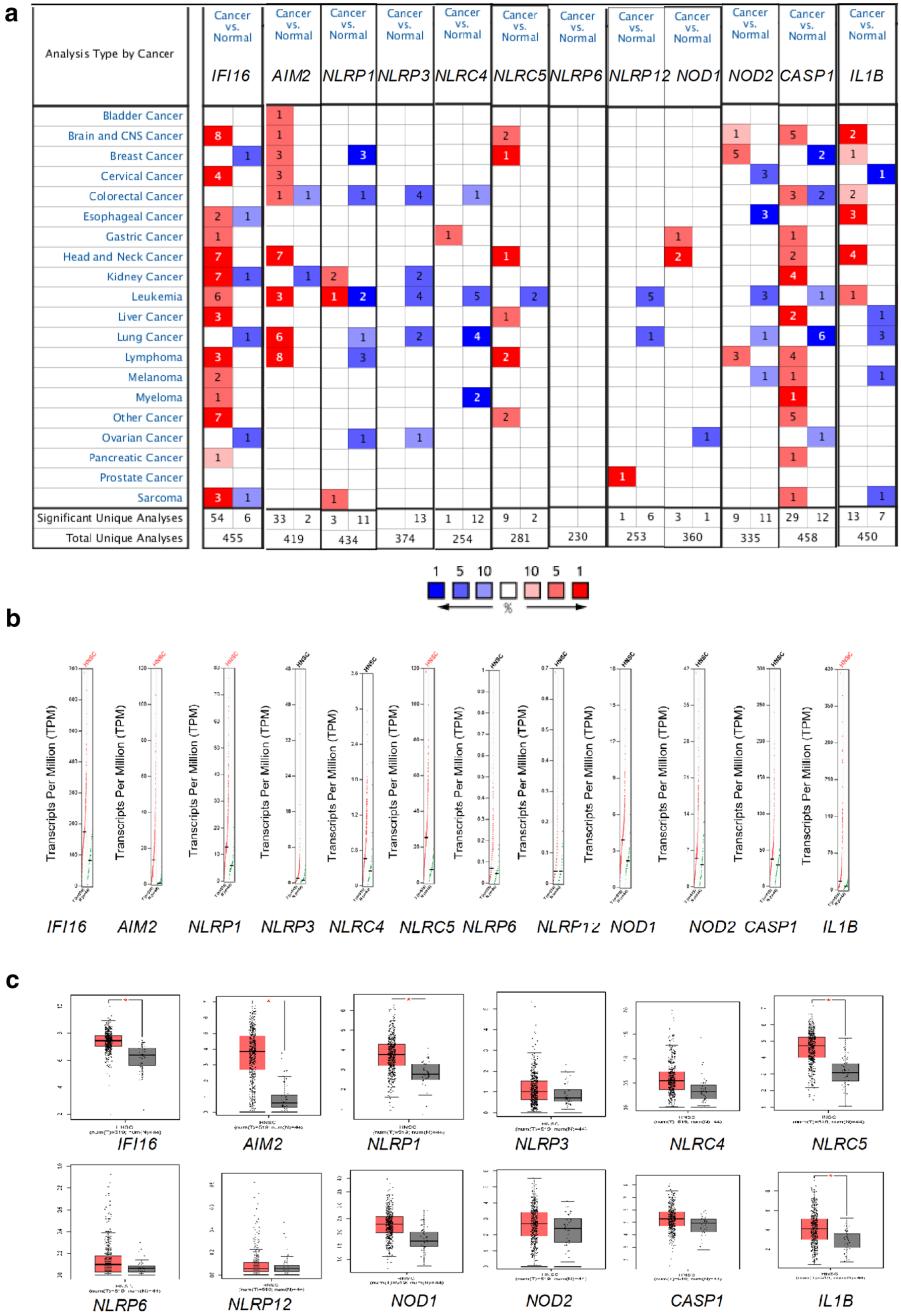
Gene expression and transcription levels of inflammasome sensors in the HNSCC patients. **a** Oncomine analysis of the transcription levels of 12 inflammasome molecules in different types of cancers. This figure indicates the numbers of datasets with statistically significant inflammasome mRNA upregulation (red) or downregulation (blue) in different types of cancers versus the corresponding normal tissues (Threshold settings: P value, 0.05; fold change, 2; gene rank, top 10%). The numbers in the colored cells represent the numbers of dataset meeting the threshold. **b**, **c** Different expressions of these inflammasome molecules between tumor and adjacent normal tissues from HNSCC in the GEPIA database. ‘*’ indicates statistical significance with *P *< 0.05. *T* tumor, *N* normal

Furthermore, the transcriptional levels of 12 genes in cancers were compared with those in normal samples by using Oncomine database (Fig. 1a). And it was found that IFI16 and AIM2 were significantly upregulated in the patients with cancer (Fig. 1a).

The mRNA expression of 12 inflammasome molecules was compared between HNSCC and the control tissues using GEPIA (Gene Expression Profiling Interactive Analysis) database (http://gepia.cancer-pku.cn/). The results indicated that the expression levels of 12 inflammasome molecules were higher in HNSCC tissues than those in normal tissues. Furthermore, the expression levels of *IFI16*, *AIM2*, *NLRP1*, *NLRC5*, and *IL1B* were significantly upregulated (Fig. 1b, c). As the PYHIN inflammasomes showed the greatest and the most significant upregulation in HNSCC patients, for our further investigation, we focused on the 2 most described PYHIN inflammasome sensor subtypes of AIM2 and IFI16 in the tissues from HNSCC patients and the controls.

Oncomine was used to further analyze the coexpression of mRNA of inflammasome pathway molecules such as IFI16, AIM2 and IL1B in HNSCC. The results showed that the co-expression index of IFI16 (0.739) in HNSCC was higher than that of other molecules such as AIM2 (0.699) and IL-1β (0.611) in the inflammasome pathway.

### Positive correlation between the expressions of IFI16 and caspase-1 in laryngeal carcinoma

**Fig. 2:**
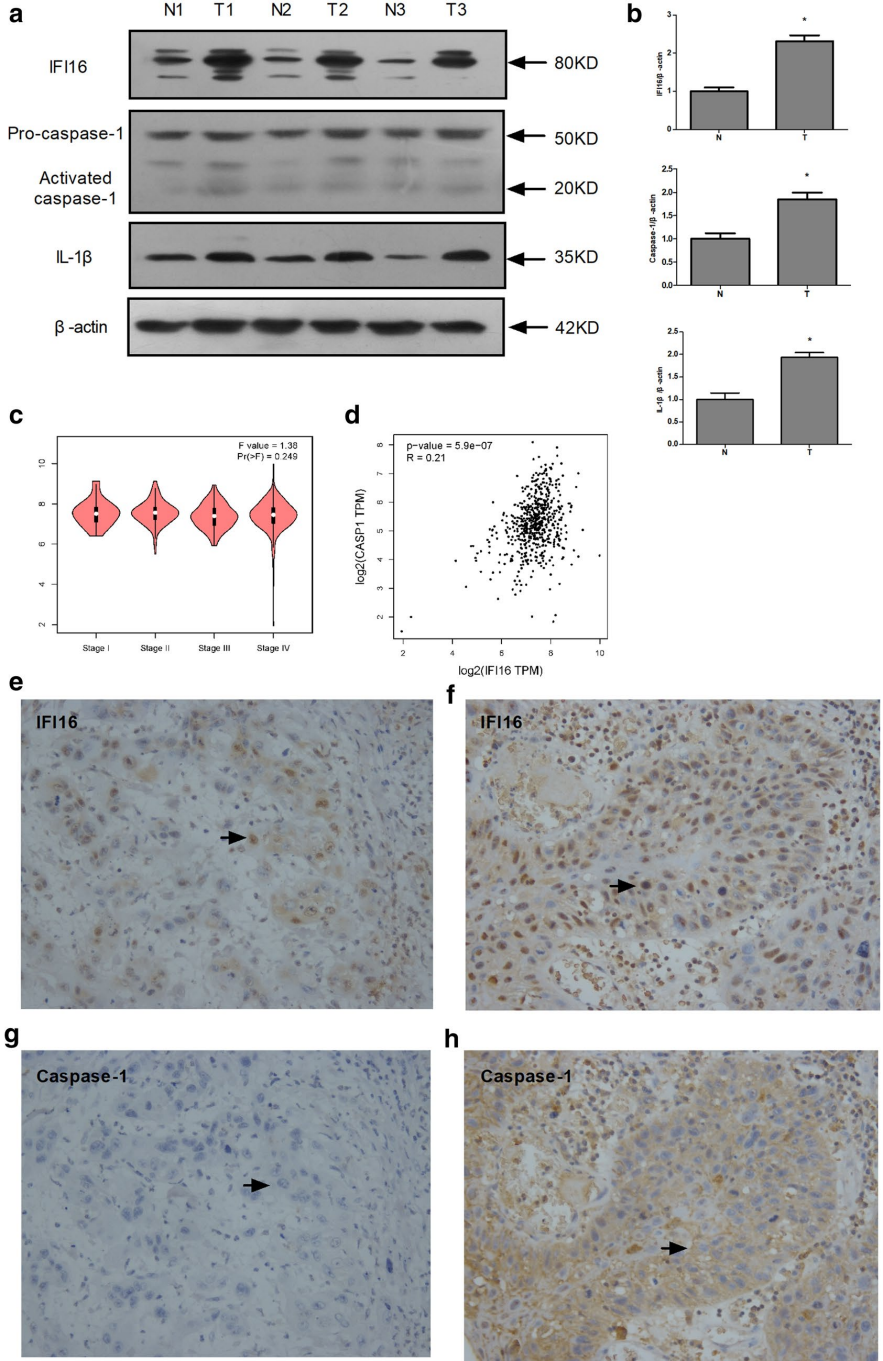
Positive correlation between the expression of IFI16 and caspase-1 in laryngeal carcinoma. **a** The protein expression of IFI16, caspase-1, IL-1β in 3 pairs of laryngeal carcinoma and adjacent normal tissues from 3 LSCC patients. N and T are used to represent adjacent and cancer tissues, respectively. β-actin served as the loading control. **b** Statistical analysis of IFI16 inflammasomes. Data were shown as mean ± SD (n = 3). ‘*’ represents that *P *< 0.05 vs. adjacent normal tissues. **c** IFI16 mRNA expression in different TNM stages in HNSCC patients (GEPIA). **d** The correlation between IFI16 mRNA expression and Caspase-1 in HNSCC patients in the GEPIA database. E-H. IFI16 and caspase-1 expression is analyzed via immunohistochemistry for 36 biopsy specimens of laryngeal carcinoma tissues. **e** IFI16 exhibited weak nuclear staining in 8 of the 36 (22.2%). **f** IFI16 exhibited strong nuclear staining in 28 of the 36 specimens (77.8%). **g** Caspase-1 exhibited weak cytoplasmic staining in 16 of the 36 specimens (44.4%). **h** Caspase-1 exhibited strong cytoplasmic staining in 20 of the 36 specimens (55.6%). Original magnification, ×400. Scale bars, 25 μm, Black arrow

Western blot was performed to test the expression of IFI16, caspase-1 and IL-1β protein in 3 pairs of primary laryngeal carcinoma and their normal para-laryngeal tissues collected from 3 patients with laryngeal cancer after surgery. The significantly increased expression of IFI16 (2.32 ± 0.12-fold), caspase-1 (1.85 ± 0.13-fold), and IL-1β protein (1.93 ± 0.10-fold) was detected in laryngeal cancer tissues compared with that in adjacent tissues (Fig. 2a, b). The data is consistent with the previous studies in Fig. 1. These results showed that IFI16 inflammasome is more highly expressed in laryngeal cancer than in those normal tissues. Therefore, IFI16 inflammasome was selected as a predictive target on laryngeal cancer.

The production of activated caspase-1 is one of the important events in the activation of inflammasome [5]. First, the expression of IFI16 was analyzed with tumor stage for HNSCC. However, IFI16 did not significantly differ (Fig. 2c). Then, GEPIA dataset was used to analyze the correlation between IFI16 and caspase-1 in HNSCC, and it was found that the level of IFI16 was positively correlated with the level of caspase-1 (R = 0.21, *P* < 0.01) (Fig. 2d). The correlation between IFI16 and caspase-1 expression was further analyzed via immunohistochemistry for 36 biopsy specimens of laryngeal carcinoma tissues (Table 2). IFI16 and caspase-1 could be successfully and simultaneously expressed in those laryngeal and para-laryngeal epithelial tissues (Fig. 2e–h). Positive expression of IFI16 was primarily observed in the nucleus (Fig. 2e, f), and that of caspase-1 was primarily observed in the cytoplasm (Fig. 2g, h). The expression of IFI16 protein was detected in 36 of 36 (100%) samples (Table 2). Weak (Fig. 2e), moderate or strong staining (Fig. 2f) was detected in 6 (16.7%), 15 (41.7%), and 15 (41.7%) of the 36 samples, respectively. Correspondingly, the expression of Caspase-1 protein was detected in 33 of 36 (91.7%) samples. Weak (Fig. 2g), moderate and strong staining (Fig. 2h) was detected in 13 (36.1%), 5 (13.9%), and 15 (41.6%) of the 36 samples, respectively. Finally, statistical analysis revealed that the level of IFI16 was positively correlated with the level of caspase-1 (rs = 0.477, *P* < 0.05) (Table 3). These results suggested that the expression of IFI16 was specifically correlated with caspase-1-mediated pyroptosis in laryngeal carcinoma tissues.

**Table 2:** Association of IFI16 expression with the clinicopathological characteristics of patients with LSCC

Variable	All patients	IFI16 protein				*P*-value
		−	+	++	+++	
Age at surgery						*P *= 0.611 > 0.05
<60	17	0	7	3	7	
≥ 60	19	0	8	6	5	
Sex						*P *= 0.826 > 0.05
Male	31	0	13	8	10	
Female	5	0	2	1	2	
Histological grade						*P *= 0.003 < 0.05
I (well)	10	0	0	3	7	
II (moderate)	25	0	15	5	5	
III (poor)	1	0	0	1	0	
Tumor stage						*P *= 0.127 > 0.05
T status						
T1/T2	28	0	10	7	11	
T3/T4	8	0	5	2	1	
N status						*P *= 0.107 > 0.05
N0	32	0	12	8	12	
N1/N2/N3	4	0	3	1	0	
M status						
M0	36	0	15	9	12	
M1	0	0	0	0	0

**Table 3:** Correlation between expression of IFI16 and caspase-1 in the patients with laryngeal carcinoma

Capase-1	IFI16 protein			
	−	+	++	+++
−	0	0	2	1
+	0	10	5	1
++	0	2	4	2
+++	0	1	0	8
rs	0.477			
*P*	0.003			

### DHA inhibits its proliferation in Hep-2 cells

**Fig. 3:**
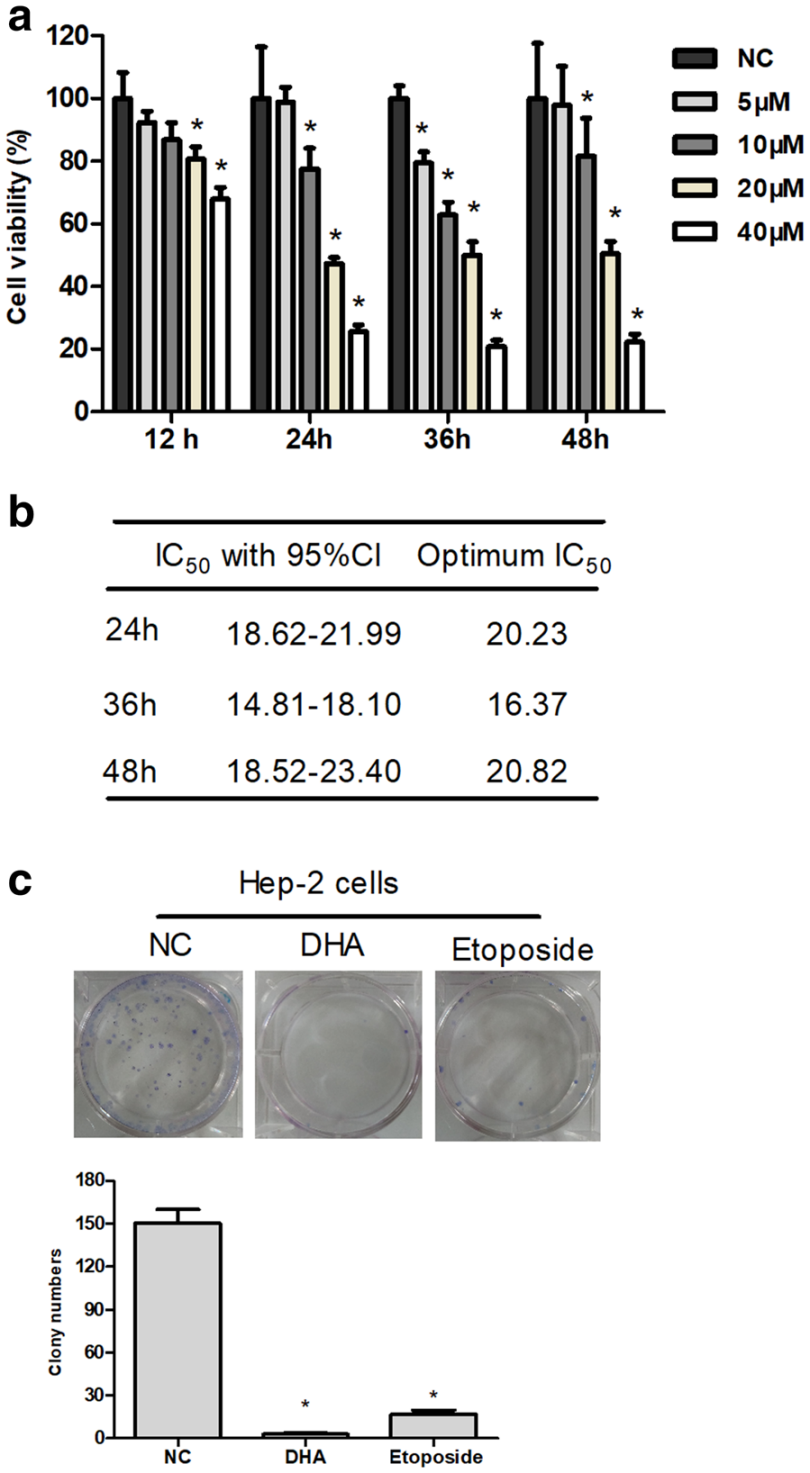
DHA inhibited cell proliferation in Hep-2 cells. **a** CCK8 was used to test the inhibitory effect of DHA on the proliferation of Hep-2 cells. Hep-2 cells were treated with DHA as indicated (mean ± SD, n= 3). **b** The IC_50_ values of cells were measured at 24, 36, and 48 h. IC_50_ values were obtained from the cytotoxicity curves using the SOFTmax PRO software. **c** Representative photographs of clonogenic assay. Hep-2 cells were treated with 20.5 μM DHA, 40 μM Etoposide, and DMSO (NC group) for 24 h, respectively. All experiments were performed for at least three times. A representative result was presented. Statistical analysis was conducted on the clone number after 14-day incubation. The inhibitory effects of growth and proliferation were calculated by number of formed cell clones. Data are shown as mean ± SD (*n *= 3). *P < 0.05 versus NC group

To test the anti-proliferative effect of DHA in vitro, human laryngeal carcinoma Hep-2 cells were respectively exposed to DHA (5, 10, 20 and 40 μM) for 12, 24, 36 and 48 h. After this treatment, cell proliferation and cytotoxicity assay (CCK-8) was conducted to assess cell viability. It was shown that DHA with greater concentrations inhibited the growth of Hep-2 cells more significantly, and its inhibition rate also increased as time went on (Fig. 3a). The result suggested that DHA cytotoxicity was dose- and time-dependent in Hep-2 cells. However, DHA showed less inhibitory effect at 12 h compared to that at any other separate time points (Fig. 3a). Hence, 24 h treatment was the optimal overtime, with a 50% inhibiting concentration (IC50) of 20.23 μM (Fig. 3b). Next, clone formation was assayed with 20.5 μM DHA for 24 h, to determine whether DHA affected the ability of long-term colony formation. Meanwhile, Hep-2 cells were treated with 40.0 μM etoposide, DNA double-strand break (DSB) agent, for 24 h and used for the positive control. It was observed that DHA-treated cell number of surviving colonies was also markedly decreased, similar to the results of Etoposide treatment (Fig. 3c). Taken together, these results suggested that DHA significantly inhibited the growth and proliferation of Hep-2 cells in dose- and time-dependent manners in vitro.

### DHA significantly reduced the expression of IL-1β in serum and xenograft tumor microenvironment

**Fig. 4:**
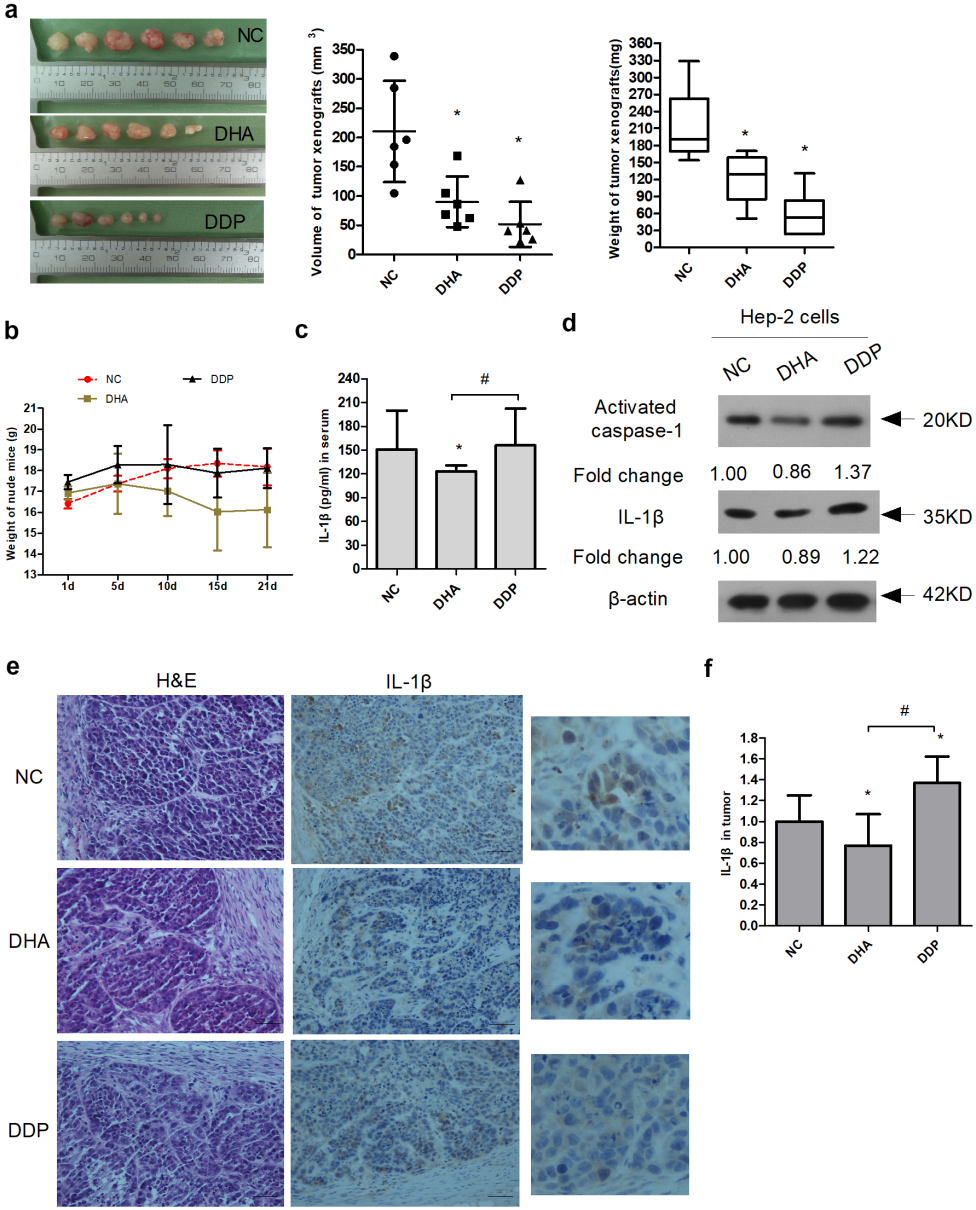
DHA significantly reduced the expression of IL-1β in serum and xenograft tumor microenvironment. **a** DHA noticeably inhibited the growth of Hep-2 xenograft tumor. Nude mice were inoculated with 1 × 10^7^ of Hep-2 cells. When the formed tumor was palpable, the mice were randomly divided into four groups. The drug treatments were carried out according to the method section. The volume, weight and diameter of tumor xenografts were presented when the mice were sacrificed. Tumor volume was calculated by the formula: V (mm^3^) = width^2^ × length × 0.5. Data were shown as mean ± SD, *P < 0.05 vs. control. **b** The body weight changes of tumor-bearing mice at the 1st and 21st days. **c** IL-1β in the serum of nude mice was measured at the 21st day. **d** Statistical analysis of caspase-1 and IL-1β expression by Western blot. Data were shown as mean ± SD (n = 3). ‘*’ represents that *P *< 0.05 vs. NC group. **e** Histological findings of the tumor were determined by H&E staining (left panels) and the expression of IL-1β protein was analyzed by immunohistochemistry (right panels) when the tumor-xenograft mice were sacrificed. Magnification ×400. **f** Expression analysis of IL-1β protein by immunohistochemistry in nasopharyngeal carcinoma tissues

The anti-tumor effect of DHA was further determined in the nude mice bearing Hep-2 tumor xenograft model. DHA (25 mg/kg/day) was administered by intraperitoneal injection for 21 days. The treatment effect was evaluated through the measurement of tumor volume. On average, DHA inhibited the tumor growth by 56.58% (Fig. 4a). On Day 21, the mice were sacrificed and the tumor weights and volumes were measured. As expected, the weight and volume of xenograft tumor were significantly reduced in the DHA-treated mice (90.11 ± 43.42 mm^3^, 121.60 ± 43.25 mg) compared with those in the controls (210.40 ± 86.51 mm^3^, 212.92 ± 63.47 mg) (Fig. 4a). These results indicated that DHA noticeably inhibited the growth of Hep-2 xenograft tumor in vivo. Meanwhile, the changes in body weight were not obvious in the tumor-bearing mice from the 1st to 21st days (Fig. 4b). Therefore, these results showed that DHA significantly inhibited the growth of xenograft tumor in nude mice.

Higher level of IL-1β in peripheral blood mononuclear cell (PBMC) cultures isolated from venous blood in the patients with laryngeal carcinoma is associated with lower 3-year and 5-year survival [25]. When the level of IL-1β was measured in serum, it was detected that IL-1β expression was significantly depressed in DHA-treated mice (123.30 ± 7.39 pg/ml) compared to that in the NC group (150.74 ± 49.63 pg/ml), and that IL-1β expression was not distinct in DDP (156.33 ± 46.18 pg/ml) (Fig. 4c). In short, DHA reduced IL-1β secretion in the serum from nude mice with xenograft tumor.

Therefore, the effect of DHA on the inflammatory microenvironment of laryngeal cancer was investigated in vivo. Western blot analysis showed that cisplatin treatment increased the expression of activated caspase-1 and IL-1β, while DHA decreased it compared with the NC group (Fig. 4d). Meanwhile, DHA decreased the expression of activated caspase-1 and IL-1β compared with that in cisplatin group (Fig. 4d). These results suggested that DHA significantly inhibited the inflammasome activation of xenograft tumor in nude mice.

Furthermore, cytotoxic and commonly prescribed chemotherapeutic drugs (for example, cisplatin, etoposide, doxorubicin) induced IL-1β secretion in primary mouse macrophages [26]. The analysis of IL-1β protein expression by immunohistochemistry in xenograft tumor showed that the expression of IL-1β protein was mainly localized to the cytoplasm in the xenograft Hep-2 cells (Fig. 4e). The result suggested that Hep-2 cells secreted IL-1β in the xenograft tumor microenvironment. Consistent with the above results in Fig. 4d, it was found that DDP promoted IL-1β production in the Hep-2 cells in vivo (Fig. 4f). Further, DHA significantly reduced the expression of IL-1β in xenograft tumor microenvironment (Fig. 4f).

### DHA suppresses the activation of IFI16 inflammasome and promotes autophagy in vitro

**Fig. 5:**
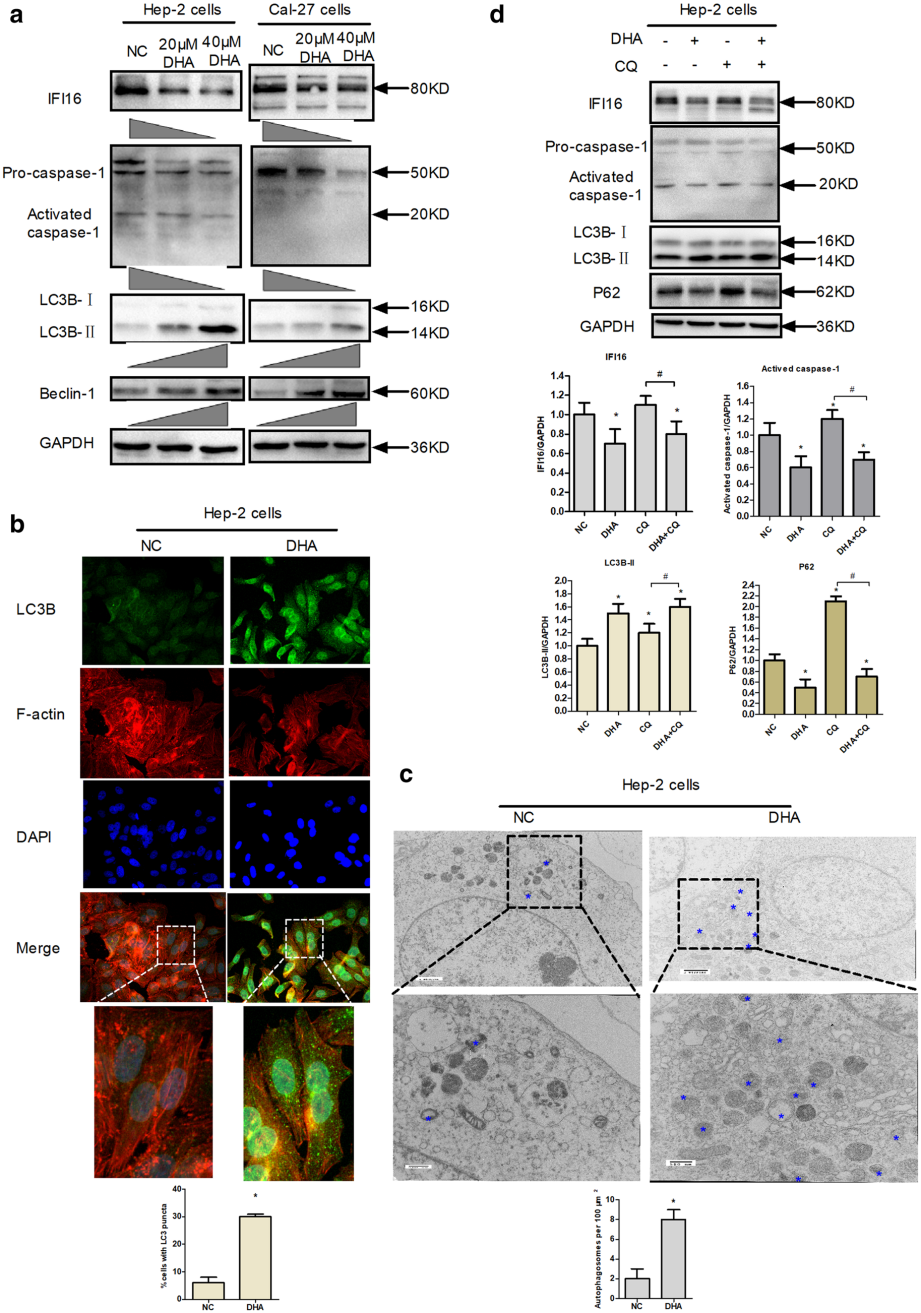
DHA suppressed the activation of IFI16 inflammasome and promoted autophagy in vitro. **a** Western blot was used to detect the expression of IFI16‐mediated inflammasome and LC3B in Hep-2 and Cal-27 cells. Cells were treated as described above. GAPDH was used as the loading control. **b** DHA-induced autophagosomes were detected in Hep-2 cells by immunofluorscent staining of LC3B (green) (×1000). Hep-2 Cells were treated with DHA (20.2 μM) and 0.1% DMSO (NC), respectively for 24 h. F-actin (red) was stained with phalloidine (red). Nuclei were counter-stained with DAPI (blue). The upper and bottom panels were respectively ×400 and ×1000. The bar chart in the lower right corner was shown as the result of statistical analysis of the autophagosome number. A total of 200 cells were counted for each group. Data were shown as mean ± SD (*n *= 3). *P < 0.05 vs. NC group. **c** The formation of autophagic vacuoles by DHA was observed under transmission electron microscope. The autophagic vacuole was indicated by blue asterisks. A total of 20 cells were counted for each group. **d** The expression of IFI16-mediated inflammasome, LC3B and p62/SQSTM1 was detected by Western blot. Hep-2 cells were exposed to 20.5 μM DHA and/or 50 μM Chloroquine (CQ; Sigma, USA) for 24 h. Data were expressed as mean ± SD (*n *= 3). *P < 0.05 vs. NC group. ^#^*P *< 0.05 vs. CQ group

The expression of IFI16 inflammasome in laryngeal cancer was further examined. As expected, DHA treatment reduced the expression levels of IFI16 and pro-caspase-1 in Hep-2 and cal-27 cells (Fig. 5a). These showed that DHA (20 and 40 μM) suppressed IFI16 inflammasome in the level of protein expression (Fig. 5a, d). In addition, DHA with high concentration (40 μM) showed strong inhibitory effect on the expression of IFI16 inflammasome. Meanwhile, DHA reduced the expression level of activated-caspase-1 in Hep-2 cells, but did not in Cal-27 cells (Fig. 5a, d). Therefore, it was suggested that DHA inhibited the activation of IFI16 inflammasome in a concentration-dependent manner in laryngeal cancer Hep-2 cells.

Western blot analysis showed that DHA promoted the conversion of LC3-I to LC3-II (Fig. 5a, d), and that the expression of LC3-II was positively correlated with DHA concentration (20 and 40 μM). Moreover, DHA increased the expression level of Beclin-1 in a dose-dependent manner (Fig. 5a). Then, autophagosomes and green fluorescent puncta under fluorescence microscopy were observed by immunofluorescent staining with LC3B antibody. The result showed a significant increase in the number of autophagosomes in DHA-treated cells compared with that in the NC group (Fig. 5b). In order to further demonstrate the morphological induction of autophagy in DHA-treated cells, ultrastructural analysis was performed with transmission electron microscopy according to the test standard for autophagosomes. The formation of double- and multiple-membrane encapsulated components (autophagosomes) was noted in the cytoplasm. Consistent with the results of Fig. 5a, b, d, the number of autophagosomes was higher in the DHA-treated group than that in the control group (Fig. 5c). These results showed that DHA induced autophagosomes in Hep-2 cells.

Furthermore, the effects of DHA on autophagy flux were examined by co-treatment of DHA with chloroquine (CQ), an autophagy flux inhibitor. The expression of p62/SQSTM1, a monitor for autophagy flux, was obviously decreased in DHA-treated Hep-2 cells compared to that in NC group and DHA + CQ group (Fig. 5d). Meanwhile, the levels of LC3B‐II increased significantly in DHA + CQ treated cells compared with those in the CQ group (Fig. 5d). These results indicated that DHA promoted autophagosome formation and restored the autophagy flux blocked by CQ in Hep-2 cells. Interestingly, the levels of IFI16 and activated-caspase-1 decreased significantly in DHA + CQ treated cells compared with those in the CQ group and NC group (Fig. 5d). The result suggested that DHA triggered IFI16/caspase-1 inflammasome suppression because of autophagy induction.

### Connecting the inflammasome and autophagy pathways

**Fig. 6:**
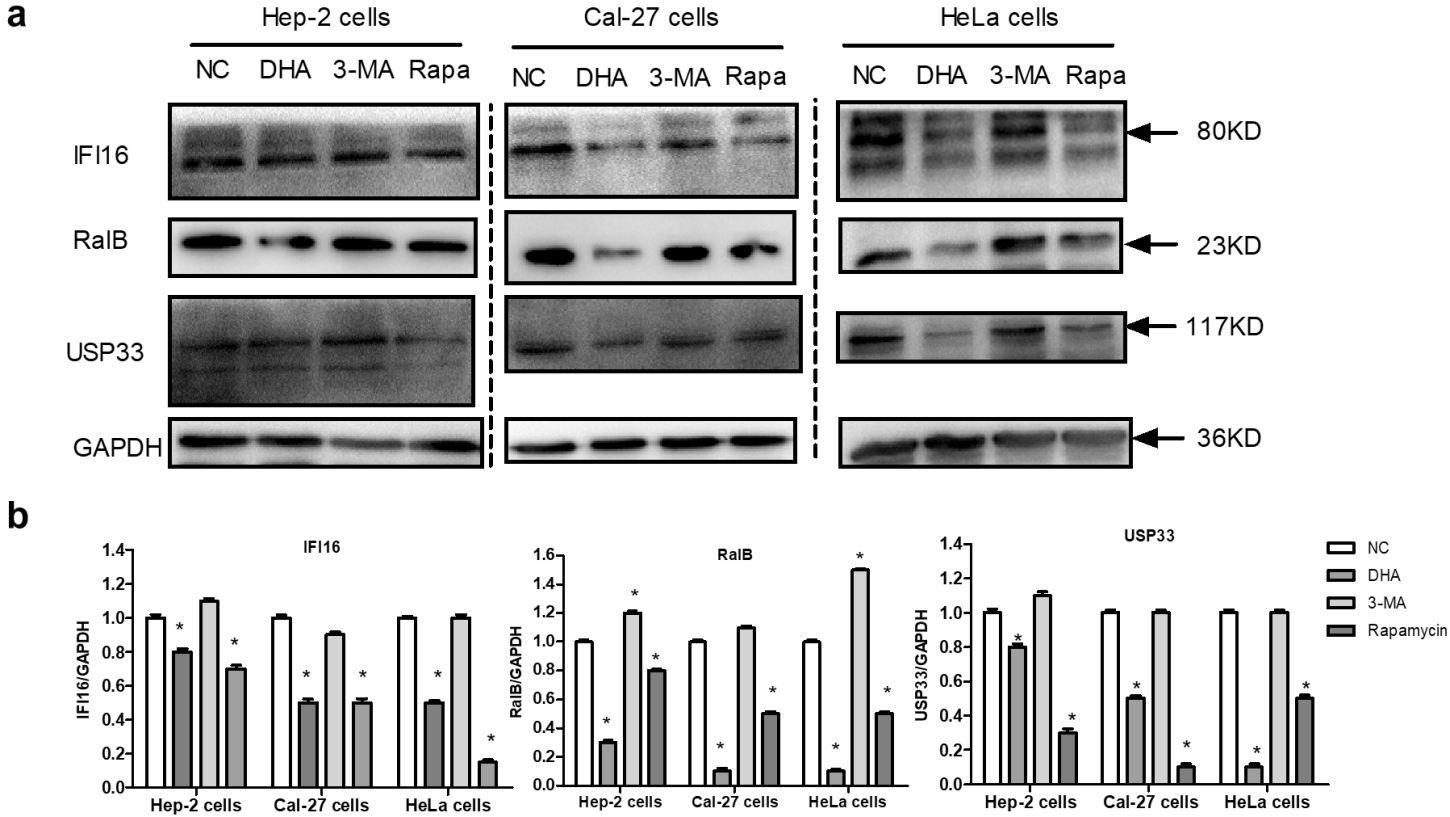
Connecting the inflammasome and autophagy pathways. **a** Western blot was used to detect the expression of IFI16 and RalB in Hep-2, Cal-27 and HeLa cells. GAPDH was used as the loading control. Hep-2, Cal-27 and HeLa cells were treated with DHA (20.2 μM, 24.5 μM, 31 μM), and 0.1% DMSO (NC) respectively for 24 h. Rapamycin (0.1 μM) acted as the autophagy activator, and 3-MA (1 mM) acted as the autophagy inhibitor. **b** Statistical analysis of IFI16, RalB and USP33. The cells were treated as described above. Data were shown as mean ± SD (n = 3). ‘*’ represents that *P *< 0.05 vs. NC

Autophagy induction is not dependent upon caspase-1 in macrophages, but upon the inflammasome sensor [6]. To test whether autophagy may affect the activity of IFI16 inflammasome in Hep-2, Cal-27 and HeLa cells, the expression level of IFI16 was examined in which autophagy was blocked using the PI3K inhibitor of 3-MA or enhanced by the mTOR inhibitor of rapamycin (Fig. 6a). Following rapamycin treatment, it was detected by immunoblotting that rapamycin induced a significant decrease of IFI16 in three cell lines (Fig. 6a, b). However, the modest amount of IFI16 was increased by blocking autophagy with 3-MA (Fig. 6a, b). These results suggested that when autophagy was activated or blocked, the amount of IFI16 was decreased or increased. Similar to rapamycin treatment, DHA reduced the expression level of IFI16 in the above three cancer cells (Fig. 6a, b). Altogether, these results indicated that the exposure to DHA triggered autophagy, which limited the IFI16 inflammasomes in cancer cells.

The activation of AIM2 or NLRP3 inflammasomes triggered RalB activation and autophagy in macrophages [6]. Next, the expression levels of RalB were measured in the Hep-2, Cal-27, and HeLa cells treated with 3-MA, rapamycin or DHA to investigate whether DHA could regulate the RalB expression (Fig. 6a). Consistent with the fractionation data of IFI16, the expression level of RalB was decreased compared to the control by augmenting autophagy through rapamycin treatment (Fig. 6a, b). However, the modest amount of RalB was increased by blocking autophagy with 3-MA (Fig. 6a, b). These results indicated that autophagy was negatively correlated with the expression level of RalB. In addition, DHA, like rapamycin, reduced the RalB expression (Fig. 6a, b).

The ubiquitin-specific processing protease 33 (USP33), as a deubiquitinating enzyme, controls the ubiquitylation of RalB GTPase at Lys 47 [8, 27]. Consistent with the data of RalB, the expression level of USP33 was decreased by DHA or rapamycin treatment, while did not change with 3-MA (Fig. 6a, b). These results suggested that enhanced autophagy can inhibit the expression of USP33.

## Discussion

**Fig. 7:**
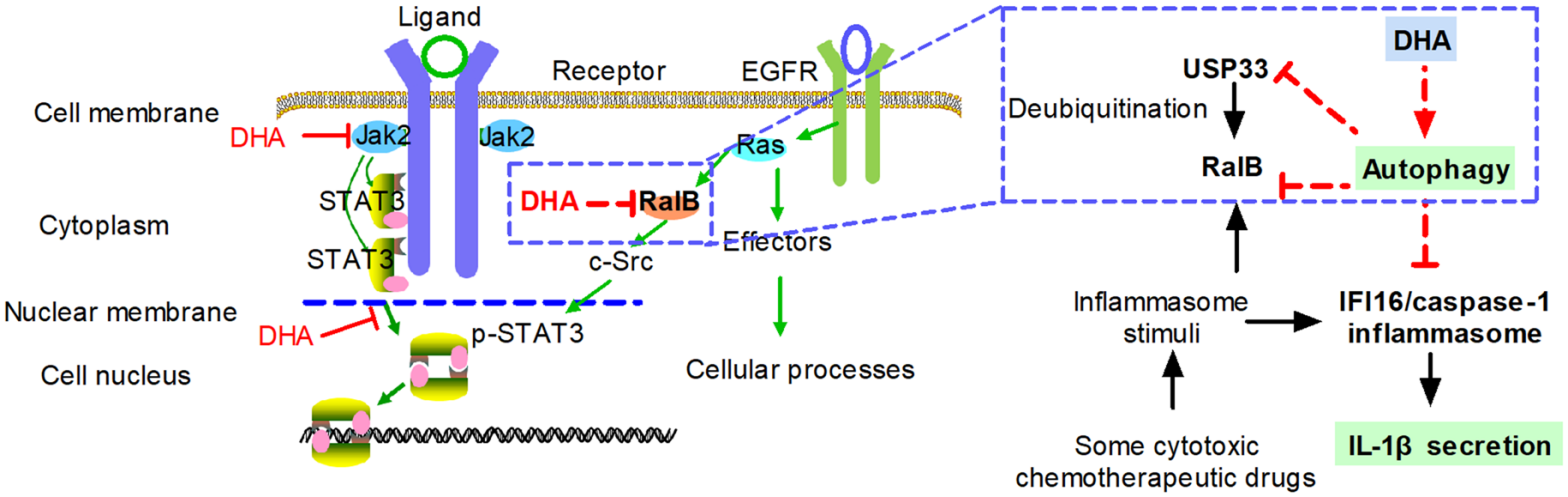
The model of the crosstalk between IFI16/caspase-1 inflammasome and autophagy by DHA in the human laryngeal squamous cell carcinoma. Mechanically, DHA triggered autophagy. Meanwhile, autophagy reduced the expression of RalB and USP33. Furthermore, DHA promoted autophagy, which suppressed the activation of IFI16 inflammasome and the secretion of IL-1β

IL-1β repression is needed to reduce inflammation in the patients under chemotherapy. The present study showed that DHA, an anti-malarial drug, significantly reduced IL-1β production in the xenograft tumor microenvironment of Hep-2 cells in nude mice. Mechanistically, DHA triggered autophagy, resulting in the down-expression of RalB and USP33. Meanwhile, enhanced autophagy suppressed the IFI16 inflammasome activation and IL-1β production (Fig. 7).

IFI16 is a scaffold protein for protein-DNA and protein–protein interactions with transcription factors. Most studies have focused on the effects of IFI16 on the growth and migration of tumor in HNSCC tissue or cell lines. It was reported that there was a strong positive correlation between IFI16 and p-STAT3 in HPV-positive lesions in 224 head and neck precancerous and malignant lesions by immunohistochemistry and FISH analysis [28]. IFI16 is a downstream regulatory molecule of p-STAT3 [29]. Our previous research also confirmed that DHA decreased p-STAT3 (Tyr705) nuclear localization in the Cal-27 cells [18]. Recently, it was found that IFI16 could act as a DNA sensor in the surveillance of cytosolic double-stranded DNA (dsDNA) to trigger the innate immune response [30]. The DNA from diverse microbes and host DNA leaking out from the mitochondria or the nucleus acted as a danger signal in damaged cells [31]. Furthermore, the DNA sensor IFI16, as a pattern recognition receptor (PRR), has the ability to sense and bind Etoposide-induced damaged self-dsDNA in the nucleus in human HaCaT keratinocytes [32]. The production of Caspase-1 is one of the important events of inflammasome activation [5]. Z-VAD-FMK, Caspase-1 inhibitor, blocked T cell apoptosis in HNSCC [33]. Inflammasome inhibitor has broad prospects in tumor therapy [34]. In line with this, the study firstly reported that IFI16 was correlated with caspase-1 in laryngeal cancer. Consistent with our results, primary effusion B-cell lymphoma cells showed that IFI16 mediated caspase-1 inflammasome activation [35]. Our study also found that DHA reduced the expression level of IFI16 in three human cancer cells of Hep-2, Cal-27, and HeLa. Further research found that DHA could also reduce the expression of pro-caspase-1 and inhibit the activation of caspase-1 in Hep-2 cells in vitro and IL-1β that was produced in tumor microenvironment in vivo. It is concluded that DHA can inhibit the expression and/or activation of IFI16 inflammasome in laryngeal cancer cells.

It is clear that chemotherapeutic drugs can promote the secretion of important pro-inflammatory cytokines such as IL-1β from monocytes and macrophages [26]. In tumor microenvironment, IL-1β contributes to epithelial-mesenchymal transition, which is related to drug tolerance in HNSCC [36]. For example, the suppression of IL-1β secretion by inhibiting inflammasome is expected to become a new treatment strategy in melanoma [37]. In addition, assembled inflammasomes can activate caspase-1 and cause IL-1β to be secreted from cancer cells [38]. In the present study, it was first found that in Hep-2 cancer cells, caspase-1 was involved in the processing and secretion of IL-1β by DDP treatment in vivo. DDP is a widely used chemotherapy drug for the clinical therapy of advanced and recurrent and/or metastatic laryngeal cancers. Furthermore, it was found that DDP increased the activation of caspase-1 and the production of IL-1β in Hep-2 cells in xenograft mice. Inflammasomes play different roles in various tissues and cells. The assembly of inflammasome activates caspase-1 and promotes cell death by pyroptosis in macrophages [5]. However, the animal model experiment of 4NQO-induced rat oral cancer proved that IL-1β changed tumor microenvironment and inhibited cell proliferation in gene silenced (LV-shIL-1β) tumor cells [39]. Moreover, 5-Fluorouracil (5-FU)-based clinical chemotherapy increased the expression and activation of NLRP3 inflammasome in oral squamous cell carcinoma (OSCC) tissues, which then mediated the chemoresistance [40]. Meanwhile, the high expression level of NLRP3 inflammasome suggested poor prognosis in 121 LSCC tumor tissues [41]. Because inflammasome plays duplex roles in cancer development, targeting inflammasome has become a novel strategy for cancer treatment [42]. For example, the inhibition of NLRP3 inflammasome/IL-1β signaling pathway may help 5-FU-based adjuvant chemotherapy of OSCC [40]. Autophagosomes transport cytoplasmic constituents to lysosomes for degradation. Here it is shown that DHA triggered the formation of autophagosome, but inhibited the activation of IFI16 inflammasomes in vitro. The induction of autophagy does not depend on the presence of the caspase-1, but on that of inflammasome sensor [6]. In concordance with this idea, it was found that the blockage of autophagy potentiated the activity of IFI16 inflammasome, whereas the stimulation of autophagy limited its activity in three human cancer cells of Hep-2, Cal-27, and HeLa.

RalB, but not RalA, is indispensable and sufficient for the activation of autophagy [43]. Moreover, Ral-EGF signaling activates STAT3 through SRC tyrosine kinase [44]. Previous research by the research group found the elevated STAT3 and EGFR in the tumor tissues from HNSCC patients [45, 46]. Recently, we have found that high phosphorylated STAT3 (Tyr-705) levels had the worst survival rate in hypopharyngeal squamous cell carcinoma [4]. We have reported that DHA selectively downregulated the level of p-Jak2 and inhibited the growth of HNSCC [47] (Fig. 7). Further, DHA disrupted the nuclear translocation of p-STAT3 (Tyr-705) and promoted autophagy in Cal-27 cells [18] (Fig. 7). The activation of RalB promoted the formation of starvation-induced autophagosome in cervical cancer HeLa cells and in immortalized bronchial epithelial HBEC3-KT cells [43]. Various inflammasome stimuli triggered autophagy by activating RalB in macrophages [6]. In this study, it was shown that DHA reduced the expression level of RalB and USP33 in three different types of cancer cells. It is speculated that this effect of DHA may be a common phenomenon in tumor cells. Consistently, one group selectively designed RalB peptide inhibitors in that it is difficult for the Ras-Ral pathway to be disrupted by traditional medicine [48]. The RalB-specific ubiquitin ligase of USP33 is a member of the USP deubiquitinase superfamily and controls the ubiquitylation of RalB GTPase at Lys 47, which provides a regulatory switch to trigger the autophagy or innate immune response in macrophages [8]. For example, nutrient deprivation triggers the accumulation of USP33 leading to RalB deubiquitylation, which triggers the assembly of RalB-EXO84-beclin-1 complexes that initiates autophagy. In this study, autophagy inhibited the expression of RalB and USP33. DHA treatment induced autophagy leading to the reduction of RalB/USP33 expression in cancer cells.

## Conclusion

Therefore, it is proposed that DHA may act as a RalB inhibitor and definite anticancer strategy needs further investigations in laryngeal cancer. Furthermore, DHA regulates the crosstalk between autophagy and IFI16/caspase-1 inflammasome, which inhibits IL-1β production in tumor microenvironment.

## Data Availability

The datasets analyzed during the current study are available from the corresponding author on reasonable request.

## Abbreviations

IFI16: Interferon-inducible 16
DHA: Dihydroartemisinin
LSCC: Laryngeal squamous cell carcinoma
HNSCC: Head and neck squamous cell carcinoma
PYHIN: Pyrin and HIN domain-containing
AIM2: Absent in melanoma
HSCC: Human hypopharyngeal squamous cell carcinoma
dsDNA: Double strand DNA
Ral: Ras-like
TSCC: Human tongue squamous cell carcinoma
CCK-8: Cell Counting Kit-8
NLR: NOD-like receptor
OSCC: Oral squamous cell carcinoma
5-FU: 5-Fluorouracil
DDP: Cisplatin
USP33: Ubiquitin-specific processing protease 33
